# Replication stress-induced Exo1 phosphorylation is mediated by Rad53/Pph3 and Exo1 nuclear localization is controlled by 14-3-3 proteins

**DOI:** 10.1186/s13008-018-0044-2

**Published:** 2019-01-04

**Authors:** Nagaraja Chappidi, Giuseppe De Gregorio, Stefano Ferrari

**Affiliations:** Institute of Molecular Cancer Research, Winterthurerstrasse 190, 8057 Zurich, Switzerland

**Keywords:** 14-3-3, Budding yeast, DNA replication, Exonuclease-1, Phosphorylation, Pph3, Rad53, Sub-cellular localization

## Abstract

**Background:**

Mechanisms controlling DNA resection at sites of damage and affecting genome stability have been the subject of deep investigation, though their complexity is not yet fully understood. Specifically, the regulatory role of post-translational modifications in the localization, stability and function of DNA repair proteins is an important aspect of such complexity.

**Results:**

Here, we took advantage of the superior resolution of phosphorylated proteins provided by Phos-Tag technology to study pathways controlling the reversible phosphorylation of yeast Exo1, an exonuclease involved in a number of DNA repair pathways. We report that Rad53, a checkpoint kinase downstream of Mec1, is responsible for Exo1 phosphorylation in response to DNA replication stress and we demonstrate a role for the type-2A protein phosphatase Pph3 in the dephosphorylation of both Rad53 and Exo1 during checkpoint recovery. Fluorescence microscopy studies showed that Rad53-dependent phosphorylation is not required for the recruitment or the release of Exo1 from the nucleus, whereas 14-3-3 proteins are necessary for Exo1 nuclear translocation.

**Conclusions:**

By shedding light on the mechanism of Exo1 control, these data underscore the importance of post-translational modifications and protein interactions in the regulation of DNA end resection.

**Electronic supplementary material:**

The online version of this article (10.1186/s13008-018-0044-2) contains supplementary material, which is available to authorized users.

## Background

The fidelity of DNA replication is essential to maintain a stable genome. Errors occurring during replication facilitate the development of cancer [1, 2]. In budding yeast, DNA replication starts at defined sequences or origins that are distributed throughout chromosomes and where proteins of the origin recognition complex (ORC) bind upon mitotic exit [3, 4]. Timely recruitment of additional components leads to the formation of fully competent replisomes that, upon firing, move bi-directionally away from origins [5]. DNA damage represents a physical impediment to replication causing fork stall and collapse, eventually resulting in chromosome breaks and genome rearrangements [6]. To prevent this, a replication checkpoint has evolved as surveillance mechanism that controls components of the replisome [7], thus allowing to coordinate cell cycle arrest with DNA repair.

Exo1 is a DNA repair nuclease of the Rad2 family originally identified in the fission yeast *S. pombe* where its activity was shown to increase ~ fivefold during meiosis, suggesting a role for Exo1 in homologous recombination [8]. Similar observations were made in *Saccharomyces cerevisiae* and *D. melanogaster* [9, 10]. Budding yeast Exo1 was also shown to participate in the processing of DNA ends at double-strand breaks (DSB) [9], in mitotic recombination [11] as well as in end-resection at uncapped telomeres [12]. A role in mutation avoidance and mismatch correction was described for *S. pombe* Exo1 [13] and later confirmed in *S. cerevisiae*, demonstrating physical and genetic interaction between yeast Exo1 and the DNA mismatch repair (MMR) proteins Msh2 [14] and Mlh1 [15]. Both *S. cerevisiae* and human EXO1 were shown to participate in the process of nucleotide excision repair (NER) after UV irradiation [16, 17]. Additionally, human EXO1 plays an important role in the repair of DSB by HR where, in a two-step resection mechanism, it was shown to carry out the extensive resection that is necessary to generate recombinogenic structures [18, 19]. Studies conducted in *S. cerevisiae* showed redundancy between Exo1 and Rad27 in processing Okazaki fragments during DNA replication [20] and the recruitment of yeast Exo1 to stalled replication forks was shown to contribute to fork stability by counteracting fork reversal [21].

Biochemically, EXO1 catalyzes the removal of mononucleotides from the 5′-end of the DNA duplex, showing a strong preference for blunt-ended, 5′-recessed termini and DNA nicks. EXO1 can process single-stranded DNA, though less efficiently than double-stranded DNA [22–24], and displays 5′-ssDNA-flap-specific endonuclease activity but does not possess endonuclease activity at bubble-like structures [23]. The mechanism by which EXO1 acts on DNA was investigated at the molecular level in a study where the catalytic domain of EXO1 was co-crystallized with a 5′ recessed-end substrate. The data showed that, in analogy to other FEN nucleases, EXO1 first splays apart the DNA duplex inducing a sharp bend proximal to the cleavage site and then frays two nucleotides at the 5′ end, facilitating access of the catalytic site to the scissile phosphodiester bond [25].

A number of laboratories, including ours, have reported that both yeast and human EXO1 are regulated by post-translational modifications such as phosphorylation, ubiquitylation and sumoylation [26–32]. A proteome wide study aimed at identifying in vivo targets of checkpoint kinases reported S_372_ as site of phosphorylation in yeast Exo1 [33]. A study addressing the effect of telomere dysfunction on DNA resection described four additional sites of phosphorylation in yeast Exo1 that appeared to exert a negative effect on Exo1 activity [31].

In the present study, we employed Pho-Tag gel technology to resolve phosphorylated Exo1 in response to stalled replication or DNA damage and identified components of the Mec1–Rad53–Dun1 pathway as well as the phosphatase Pph3 in the dynamic control of yeast Exo1 phosphorylation. Furthermore, we explored the role of 14-3-3 proteins and Rad53 on the subcellular localization of Exo1 during checkpoint activation and recovery.

## Results

### Exo1 phosphorylation upon replication stress is Rad53-dependent and Dun1-independent

**Fig. 1:**
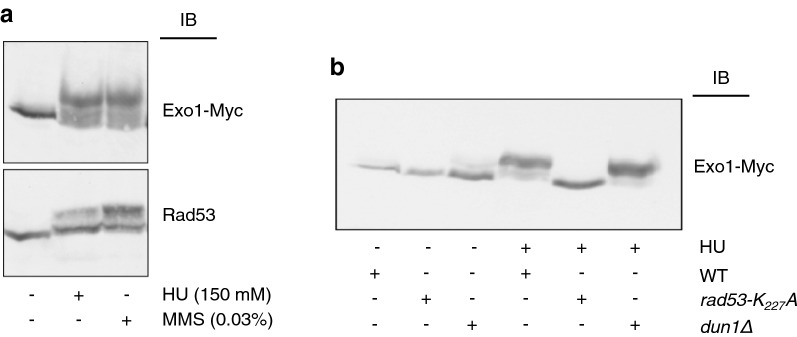
**a** Equal amounts of TCA extracts obtained from cells treated in the presence or the absence of HU (150 mM) or MMS (0.03%) for 90 min were resolved on either regular (Rad53) or Phos-Tag (Exo1-Myc) 8% SDS-polyacrylamide gels. Protein expression was examined by immunoblot (IB) with appropriate antibodies. **b** Equal amounts of TCA extracts obtained from the indicated strains treated in the presence or the absence of HU (150 mM) were resolved as in (**a**). Exo1 was visualized with an antibody to the Myc-tag

We have previously provided evidence that checkpoint-dependent phosphorylation of yeast Exo1 in response to DNA replication stress occurs in a Mec1-dependent manner and this correlates with binding to 14-3-3 proteins [30]. Taking advantage of the superior performance of Phos-Tag [30] as compared to regular SDS-PAGE [31] in resolving phosphorylated forms of Exo1, we first visualized the effect of DNA damage by an alkylating agent or of stalled DNA replication on Exo1 mobility (Fig. 1a and Additional file 1: Fig. S1). Next, we set out to dissect the contribution of Mec1 kinase cascade components to Exo1 phosphorylation. To this end, we examined the response to HU in strains expressing a hypomorphic Rad53 mutant or that are deficient in Dun1. We first confirmed that the HU-sensitivity of the former could be partially rescued by deletion of *EXO1* [30] and that cells carrying a *DUN1* deletion were less sensitive to HU than Rad53-deficient cells (Additional file 1: Fig. S2). Next, we observed that the HU-induced and phosphorylation-dependent retarded migration of Exo1 was abolished in the checkpoint defective Rad53-mutant strain (*rad53*-*K227A*), but not in *dun1Δ* cells (Fig. 1b).

These data unequivocally indicate that Rad53 is the component of the Mec1 pathway responsible for Exo1 phosphorylation in response to DNA replication stress.

### Pph3 controls Rad53 and Exo1 dephosphorylation during recovery

**Fig. 2:**
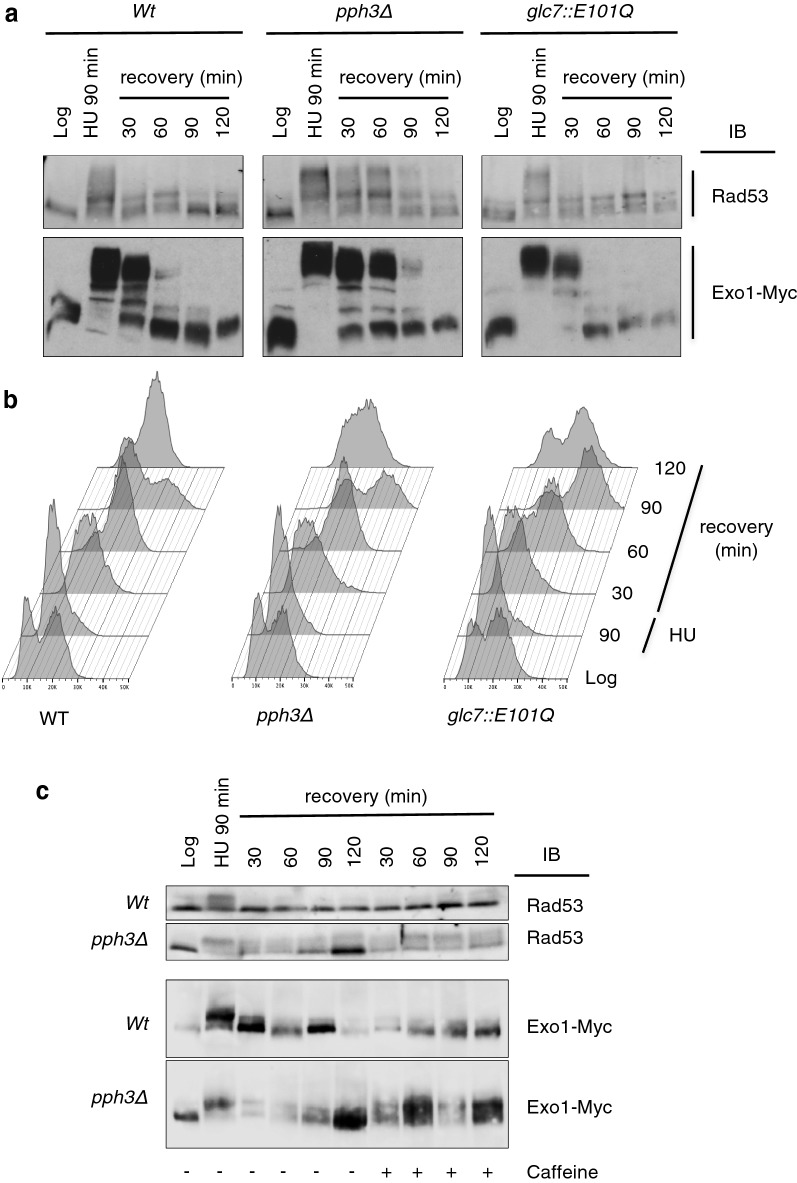
**a** Wild type, *pph3Δ* and *glc7::E101Q* strains were synchronized with HU (150 mM, 90 min) and released in YPD for the indicated times. Equal amounts of proteins were resolved as in Fig. 1. To improve the resolution of protein bands, one additional hour of separation was allowed after the die front reached the end of the slab gel. Proteins of interest were detected by immunoblot (IB). **b** Flow cytometric analysis of DNA content for the samples displayed in (**a**). **c** Wild type and *pph3Δ* strains were synchronized as described in (**a**) and released in YPD in the presence or the absence of caffeine (10 mg/ml) for the indicated times. Proteins of interest were detected as in (**a**)

In order to establish which phosphatase opposes Rad53-dependent Exo1 phosphorylation during recovery from DNA replication stress, we made use of strains deficient in Pph3 or Glc7, representative of the two major Ser/Thr phosphatase activities in the cell and previously shown to play a role in DDR [34–37]. HU treatment caused a mobility shift for both Rad53 and Exo1 that was more prominent in *pph3Δ* than in wild type cells, indicative of higher stoichiometry of phosphorylation in the former background (Fig. 2a). In *pph3Δ* cells, Exo1 remained fully phosphorylated until 60 min post-release from HU, whereas Rad53 underwent partial dephosphorylation at this time (Fig. 2a). To assess whether Pph3 acts directly on Exo1 or has an indirect effect on it through dephosphorylation of Rad53, we switched-off the checkpoint during HU recovery using the Mec1 inhibitor caffeine. The data showed that, under these conditions, both Rad53 and Exo1 dephosphorylation were delayed in *pph3Δ* cells (Fig. 2c), leaving open the possibility that Pph3 may control dephosphorylation of both Rad53 and Exo1. Flow cytometric analysis of DNA showed a delay of cell cycle progression upon HU removal in *pph3Δ* cells (Fig. 2b, 60 and 90 min time-points), which was possibly the consequence of prolonged Rad53 activation (Fig. 2a).

The established lack of viability of *glc7*-null cells [38] prompted us to take advantage of a catalytic mutant of the phosphatase (*glc7*-*E101Q*), which displays defects in glucose metabolism but normal cell cycle progression and chromosome segregation [39]. The data showed that the pattern of Exo1 or Rad53 mobility during recovery from HU in the *glc7*-*E101Q* background was not altered in comparison to control cells (Fig. 2a), ruling out a contribution from Glc7 in the dephosphorylation of these proteins during checkpoint recovery.

Taken together, these data indicate that Pph3 is the major phosphatase involved in Rad53 and Exo1 dephosphorylation during recovery from HU.

### 14-3-3 proteins control Exo1 localization

**Fig. 3:**
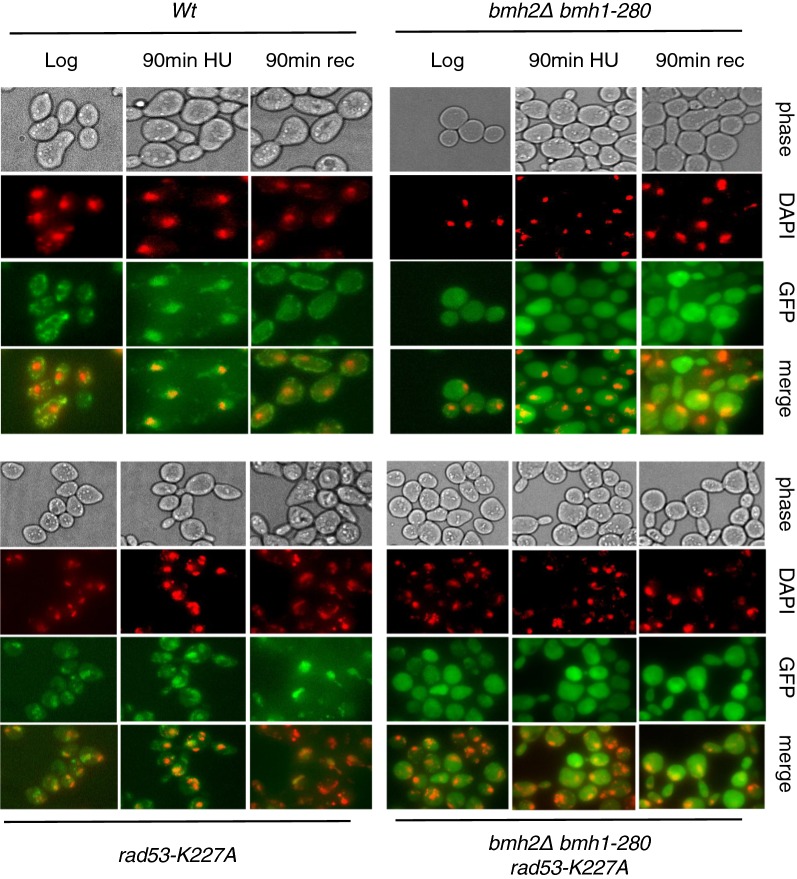
Fluorescence microscopy images of wild type and mutant strains that were treated as indicated. From top to bottom: Phase contrast, DAPI (rendered as red), Exo1-GFP (green) and a merge of DAPI and GFP. Five fields in each condition, containing ~ 30 cells/field, were examined in at least three distinct experiments

Considering our previous finding that yeast Exo1 interacts with 14-3-3 proteins upon replication stress and that such interaction prevents over-resection of DNA at, and behind, replication forks [30], we asked whether 14-3-3 proteins may exert their action through effects on Exo1 localization. To visualize Exo1, we tagged the endogenous gene with GFP and examined its localization by fluorescence microscopy. In wild type cells, we observed Exo1-GFP nuclear accumulation in response to HU-treatment. Upon release from HU, the green fluorescence signal was redistributed throughout the cell, with an apparent decrease in intensity when compared to untreated cells (Fig. 3). Next, we visualized the localization of Exo1-GFP during transition through the cell cycle in the absence of DNA damage. To this end, we synchronized wild type cells in late G1 using α-factor and released them to allow synchronous entry into S-phase. Under these conditions, we observed nuclear accumulation of Exo1 during S-phase and redistribution of the green fluorescence signal through the cell at later times (Additional file 1: Fig. S3), in a manner similar to what observed under conditions of low nucleotide availability (Fig. 3).

To examine possible effects of 14-3-3 proteins on the localization of Exo1-GFP we employed the *bmh1*-*280 bmh2Δ* double mutant strain, which is characterized by normal cell cycle progression in unperturbed conditions, but shows recovery defects in response to HU [40]. The mutant Bmh1-280 protein carries a single point mutation (E_136_ > G) in helix αE, affecting a residue close to the amino acids that form salt bridges and hydrogen bonds with the ligand [41]. In *bmh1*-*280 bmh2Δ* cells (hereafter 14-3-3-deficient cells), the intense green fluorescence signal of Exo1-GFP remained diffused in response to treatment with HU, failing to shuttle into the nucleus (Fig. 3).

**Fig. 4:**
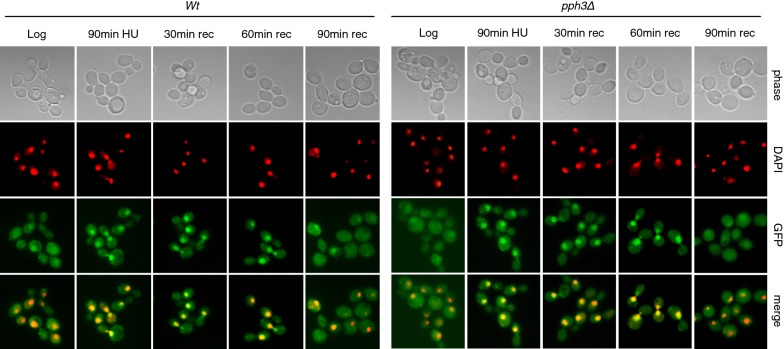
Fluorescence microscopy images of wild type and *pph3Δ* cells that were treated as indicated. From top to bottom: Phase contrast, DAPI (rendered as red), Exo1-GFP (green) and a merge of DAPI and GFP. Five fields in each condition, containing ~ 20 cells/field, were examined in at least three distinct experiments

*Rad53*-*K227A* cells express a kinase-defective Rad53 and are characterized by uncontrolled firing of dormant origins and destabilization of replication intermediates [30, 42], the latter being relieved by *EXO1* deletion [21]. In the Rad53-mutant strain undergoing replication stress, Exo1-GFP was able to accumulate into the nucleus, but failed to be displaced from it and redistribute in the cell during recovery from HU (Fig. 3). To ascertain whether the persistent nuclear accumulation of Exo1-GFP in the Rad53-mutant strain recovering from HU was due to failed phosphorylation by kinase-defective Rad53, we examined localization of Exo1-GFP in cells carrying deletion of *PPH3*, a condition where Rad53 remains partially phosphorylated during recovery (Fig. 2a). The data showed that a similar pattern of nuclear localization and release of Exo1-GFP in wild type and *pph3Δ* cells upon treatment and release from HU, respectively (Fig. 4). Finally, in *rad53*-*K227A/bmh2Δ bmh1*-*280* cells the pattern of Exo1-GFP distribution upon HU-treatment and during recovery appeared similar to the 14-3-3-deficient cells (Fig. 3).

These data suggest that 14-3-3 proteins are necessary to facilitate Exo1 nuclear translocation, whereas the Rad53/Pph3 control of Exo1 phosphorylation does not seem to play a role in the export of Exo1 from the nucleus.

## Discussion

Phosphorylation of yeast Exo1 upon checkpoint activation was so far investigated by means of cumbersome mass spectrometry and the effect of Exo1 phosphorylation on DNA resection was indirectly assessed from patterns of cell viability [31, 33]. We have previously shown the power of Phos-Tag technology, which allows resolving a number of phosphorylated Exo1 isoforms induced in response to stalled replication [30]. In the present study, combining Phos-Tag SDS-PAGE resolution of proteins and Western blot analysis (Fig. 1a and Additional file 1: Fig. S1), we confirm and extend these observations, demonstrating that Exo1 undergoes phosphorylation in response to DNA damage by an alkylating agent as well as by an agent that stalls DNA replication. With regard to the protein kinase cascade signaling to Exo1, we previously compared the pattern of Exo1 mobility in wild type and *mec1/sld1Δ* cells, concluding that retarded Exo1 migration in Phos-Tag gels depended on Mec1 activity [30]. Extending those observations, we now provide evidence for a similar retardation of migration in *rad53*-*K227A* cells, but not in *dun1Δ* cells, challenged with a replication inhibitor (Fig. 1b). This indicates that Exo1 phosphorylation in response to HU is predominantly Rad53-dependent.

The major Ser–Thr protein phosphatase activities in the cells, namely those associated with the type-1 and type-2A enzymes Glc7 and Pph3, respectively, have been shown to play a major role during recovery from DNA replication stress or MMS-induced damage through dephosphorylation of γH2AX and Rad53 [34, 36, 37]. We demonstrate that absence of Pph3 caused an evident retardation of Exo1 dephosphorylation during recovery from HU and also contributed to delay dephosphorylation of Rad53 (Fig. 2a). Checkpoint switch-off by caffeine in *pph3Δ* cells showed that dephosphorylation of both Rad53 and Exo1 was delayed (Fig. 2c), indicating that Pph3 may act directly or indirectly on Exo1.

We have previously shown that 14-3-3 proteins bind both human and yeast Exo1, with the distinction that the former establishes a constitutive interaction, whereas the latter binds 14-3-3 only upon stalled replication [30]. Hence, checkpoint-dependent phosphorylation of yeast Exo1 likely creates the conditions for interaction with 14-3-3 proteins, whereas in human EXO1 suitable sites are likely phosphorylated in a checkpoint-independent manner. In human EXO1, at least six phosphorylation sites potentially responsible for this protein–protein interaction were identified [43]. Functional analysis with phospho-null mutants indicated that they have no role in nuclear import of EXO1 [43]. A subsequent study identified the domain 508–750 of EXO1, which comprises a number of previously identified sites of phosphorylation, as the region responsible for interaction with 14-3-3 [44]. The authors showed that 14-3-3 protein binding through this region causes inhibition of EXO1-mediated DNA resection in a cell-free system but not in a biochemical resection assay with purified components, indicating the participation of yet unidentified components in this process [44]. Regardless of this, the study revealed a functional role for the interaction of 14-3-3 proteins with EXO1. Such modulatory effect of 14-3-3 proteins on the human enzyme is in agreement with our previous findings in budding yeast, where we showed that lack of functional Bmh1 and Bmh2 proteins (the yeast homologues of human 14-3-3 proteins) unleashes Exo1 activity, causing pathological resection of DNA [30]. Using wild type and mutant strains, in the present study we discovered an additional role of yeast 14-3-3 proteins, namely an effect on the dynamic shuttling of Exo1 through cell compartments. We provide visual evidence that yeast Exo1 is recruited to nuclei during regular transition through S-phase (Additional file 1: Fig. S3) as well as in response to replication stress (Fig. 3), in a manner that depends on 14-3-3 proteins. Release of Exo1 from nuclei of wild type cells occurs during the recovery phase (Fig. 3). In Rad53-deficient cells recovering from replication stress, the release of Exo1 from nuclei was impaired (Fig. 3), a fact that can be correlated to failed phosphorylation by kinase-defective Rad53. However, in *pph3Δ* cells where wild type Rad53 remained partially phosphorylated during initial phases of recovery (Fig. 2a), hence possibly able to phosphorylate Exo1, and when Exo1 itself was hyper-phosphorylated, we did not observe preferential exclusion of Exo1 from the nucleus (Fig. 4), indicating that the Rad53/Pph3 control of Exo1 phosphorylation does not likely play a role in nuclear export of Exo1.

Interestingly, in the absence of functional 14-3-3 proteins, we observed that Exo1 remained distributed in all cell compartments, regardless of the presence of HU (Fig. 3), with the nuclear sub-population of Exo1 likely being the one responsible for the reported over-resection of DNA [30]. This suggests that nuclear localization of Exo1 requires interaction with 14-3-3 proteins. Since it was previously reported that yeast 14-3-3 proteins bind to the checkpoint kinase Rad53, influencing its DNA damage-dependent functions [45], and we showed that yeast 14-3-3 proteins bind Exo1 upon replication stress [30], it is likely that 14-3-3 proteins coordinate phosphorylation of Exo1 by Rad53.

## Conclusion

This work demonstrates that the dynamic phosphorylation of yeast Exo1 in response to stalled replication is under the control of the checkpoint kinase Rad53. During recovery from HU the phosphatase Pph3 results to be the major controller of Rad53 and Exo1 phosphorylation. Furthermore, this study extended previous observations on the interaction between Exo1 and 14-3-3 proteins showing that the latter contribute to shuttle Exo1 into the nucleus under conditions of normal DNA replication or upon stress caused by low nucleotide availability.

As a whole, this study sheds further light on the control of a key component of the DNA resection machinery.

## Materials and methods

### Materials

The antibodies used in this study were: mouse monoclonal anti-Myc (9E10, sc-40), mouse monoclonal (sc-74427) and goat polyclonal (sc-6749) anti-Rad53, rat monoclonal anti-α-tubulin (sc-53030) from Santa Cruz Biotechnology. Hydroxyurea was purchased from Bio-Basic Canada Inc., whereas α1-mating factor and all other reagents were from Sigma.

### *Saccharomyces cerevisiae* strains

The yeast strains used in this study are isogenic to W303-1A (wild type) [46] and are listed in Table 1. All strains have been obtained by one step replacement using the indicated markers and tags that have been generated by PCR. Yeast transformation was performed by LiAc/SS carrier DNA/PEG method [47]. The isolated clones have been verified by colony PCR and Western Blot. All deletion (Δ) strains lack the entire coding sequence.

**Table 1:** List of *S. cerevisiae* strains used in this study

Strain	Genotype	Origin
W303-1A	MATa leu2-3,112 trp1-1 can1-100 ura3-1 ade2-1 his3-11,15 [phi+]	[46]
YCN51	MATa EXO1-Myc::KANMX4::exo1	This study
CY2034	MATa rad53-K227A::KANMX4	[21]
CY5469	MATa rad53-K227A::KANMX4 exo1Δ::HIS3	[21]
YCN68	MATa EXO1-Myc::KANMX4::exo1 dun1Δ::URA3	This study
YCN117	MATa EXO1-Myc::KANMX4::exo1 pph3Δ::URA3	This study
YCN57	MATa glc7-E101Q::KANMX4 EXO1-Myc::NAT1::exo1	This study
YKE2	MATa bmh2Δ::NAT1 bmh1Δ::HIS3::bmh1-280::LEU2	[30]
YKE8	MATa bmh2Δ::NAT1 bmh1Δ::HIS3::bmh1-280::LEU2 exo1::URA3 rad53-K227A::KANMX4	[30]
YKE37	MATa exo1Δ::KANMX6	[30]
YCN44	MATa EXO1-GFP::KITRP1-1::exo1	This study
YCN45	MATa bmh2Δ::NAT1 bmh1Δ::HIS3::bmh1-280::LEU2 EXO1-GFP::KITRP1-1::exo1	This study
YCN46	MATa rad53-K227A::KANMX4 EXO1-GFP::KITRP1-1::exo1	This study
YCN47	MATa rad53-K227A::KANMX4 bmh2Δ::NAT1 bmh1Δ::HIS3::bmh1-280::LEU2 EXO1-GFP::KITRP1-1::exo1	This study
YSF169	MATa EXO1-GFP::KITRP1-1::exo1 pph3Δ::URA3	This study

### α-Factor synchronization

Log-phase cells were treated with 5 µg/ml α-factor for 120 min, released in fresh YPD containing 50 µg/ml pronase and harvested in a time-course fashion.

### Sensitivity assays

Wild-type and mutant strains were grown exponentially. Serial dilutions (1:10) were spotted on YPD plates containing different HU concentrations and grown for 3 days before scoring.

### Protein extraction and Western Blotting

Western blot analysis of yeast proteins was carried out upon TCA extraction [48]. To visualize Exo1, an optimized Phos-tag system (50–150 µM Phos-tag reagent) was employed according to [49]. Proteins were transferred to PVDF (0.45 µm pore size, Machery-Nagel) overnight at 4 °C applying constant voltage (30 V). Immunoblot analysis was performed as previously described [28] and proteins were visualized using the FUSION SOLO^®^ chemiluminescence imaging system (Vilber).

### Flow cytometric analysis

Flow cytometric analysis was performed as described [50].

### Fluorescence microscopy

Yeast cells were fixed in 4% formaldehyde and mounted with Vectashield^®^ (containing DAPI). Cells were imaged with an Olympus 1X71 fluorescence microscope. An oil immersion 100× objective was used. DAPI was rendered in red as false color.

## Additional file

**Additional file 1: Figure S1.** Exo1-Myc and Rad53 from wildtype cells that were treated as indicated were resolved on a regular 8% Laemmli SDS-polyacrylamide gel. The indicated proteins were detected by immune-blotting (IB). Tubulin was used as loading control. **Figure S2.** Spot dilution assays on YPD plates containing different amounts of HU. The indicated strains were grown for 3 days before scoring cell survival. **Figure S3.** Wildtype cells were synchronized for 120 min in α-factor, released for the indicated times and examined by fluorescence microscopy. From top to bottom: Phase contrast, DAPI (rendered as red), Exo1-GFP (green) and a merge of DAPI and GFP.
