# Supplementary material for: Replication stress-induced Exo1 phosphorylation is mediated by Rad53/Pph3 and Exo1 nuclear localization is controlled by 14-3-3 proteins
Source: Cell Div. 2019 Jan 4;14:1. doi: 10.1186/s13008-018-0044-2 (PMC6318887; doi:10.1186/s13008-018-0044-2)
Supplement: Supplementary file 1 — Additional file 1: Figure S1. Exo1-Myc and Rad53 from wildtype cells that were treated as indicated were resolved on a regular 8% Laemmli SDS-polyacrylamide gel. The indicated proteins were detected by immune-blotting (IB). Tubulin was used as loading control. Figure S2. Spot dilution assays on YPD plates containing different amounts of HU. The indicated strains were grown for 3 days before scoring cell survival. Figure S3. Wildtype cells were synchronized for 120 min in α-factor, released for the indicated times and examined by fluorescence microscopy. From top to bottom: Phase contrast, DAPI (rendered as red), Exo1-GFP (green) and a merge of DAPI and GFP. [file 13008_2018_44_MOESM1_ESM.pdf]

Replication stress-induced Exo1 phosphorylation is mediated by Rad53/Pph3 and Exo1 nuclear localization is controlled by 14-3-3 proteins

Nagaraja Chappidi, Giuseppe De Gregorio and Stefano Ferrari
Institute of Molecular Cancer Research, Winterthurerstrasse 190, CH-8057 Zurich, Switzerland

ADDITIONAL FILE

ADDITIONAL FIGURE LEGENDS

Figure S1

Exo1-Myc and Rad53 from wildtype cells that were treated as indicated were resolved on a regular 8% Laemmli SDS-polyacrylamide gel. The indicated proteins were detected by immune-blotting (IB). Tubulin was used as loading control.

Figure S2

Spot dilution assays on YPD plates containing different amounts of HU. The indicated strains were grown for 3 days before scoring cell survival.

Figure S3

Wildtype cells were synchronized for 120 min in α-factor, released for the indicated times and examined by fluorescence microscopy. From top to bottom: Phase contrast, DAPI (rendered as red), Exo1-GFP (green) and a merge of DAPI and GFP.

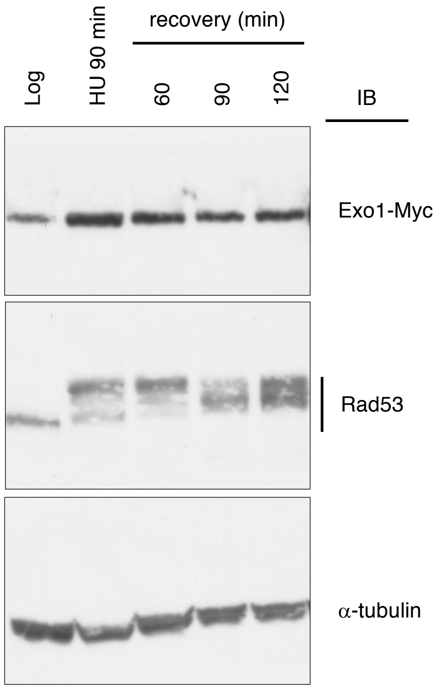

recovery (min)
Log
HU 90 min
60
90
120
IB
Exo1-Myc
Rad53
α-tubulin

Fig. S1

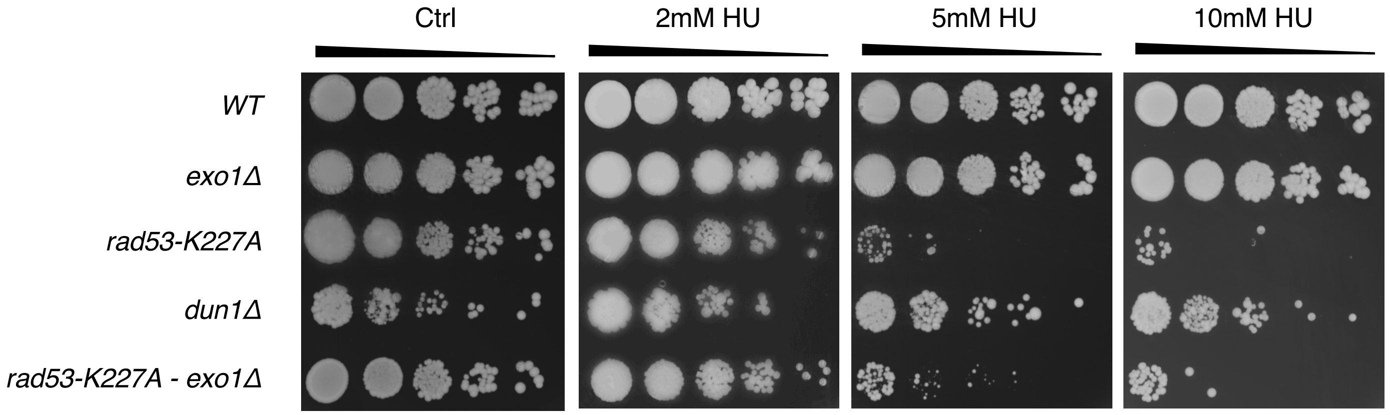

Ctrl
2mM HU
5mM HU
10mM HU
WT
exo1Δ
rad53-K227A
dun1Δ
rad53-K227A - exo1Δ

Fig. S2

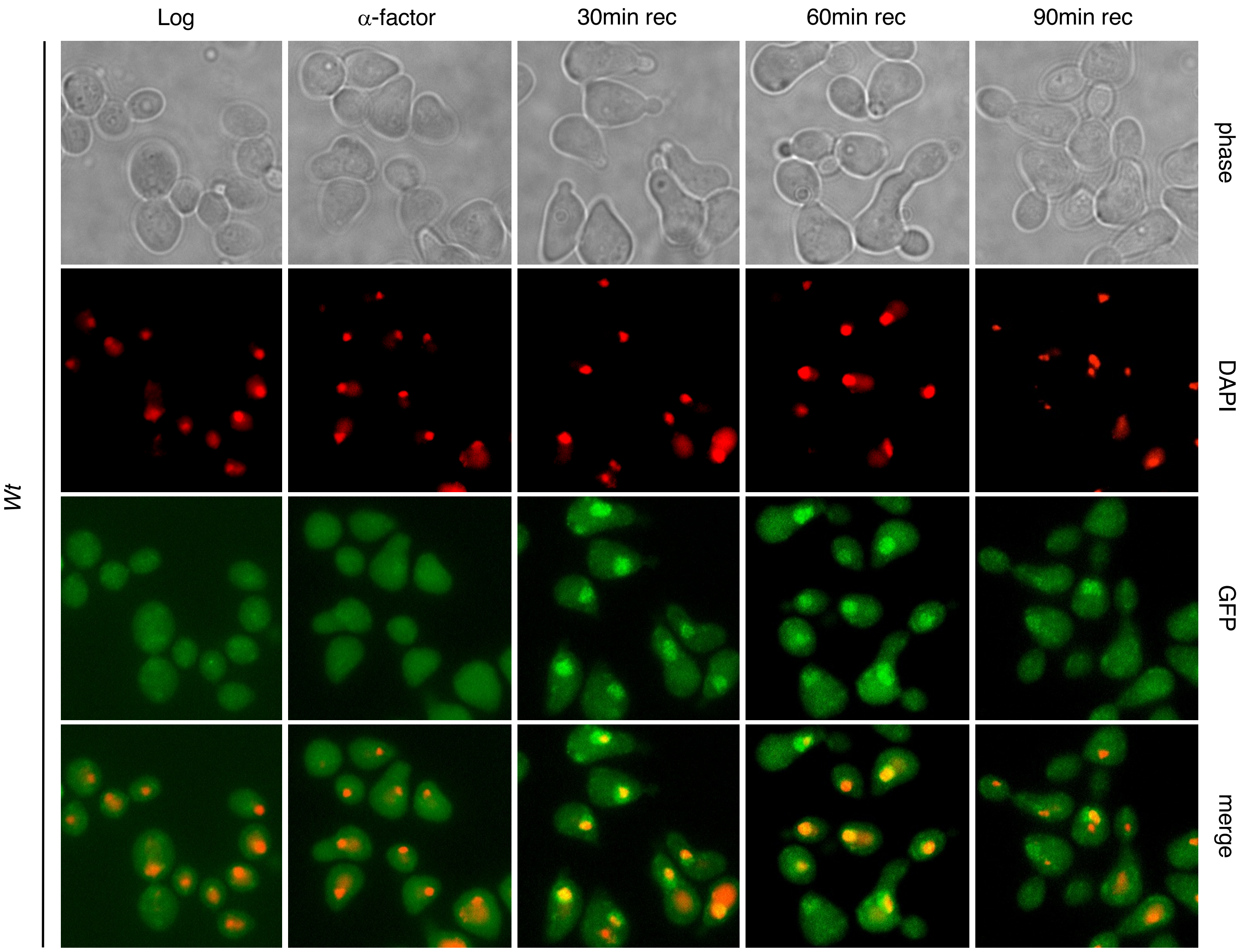
Log
α-factor
30min rec
60min rec
90min rec
phase
DAPI
GFP
merge
Wt

Fig. S3
